# Nitrogen economy of alpine plants on the north Tibetan Plateau: Nitrogen conservation by resorption rather than open sources through biological symbiotic fixation

**DOI:** 10.1002/ece3.6038

**Published:** 2020-01-27

**Authors:** Ning Zong, Minghua Song, Guangshuai Zhao, Peili Shi

**Affiliations:** ^1^ Key Laboratory of Ecosystem Network Observation and Modelling Institute of Geographic Sciences and Natural Resources Research Chinese Academy of Sciences Beijing China; ^2^ China National Forestry-Grassland Economics and Development Research Center National Forestry and Grassland Administration Beijing China; ^3^ College of Resources and Environment University of Chinese Academy of Sciences Beijing China

**Keywords:** Alpine grassland, biological nitrogen fixation, legume species, nitrogen resorption efficiency, precipitation gradient

## Abstract

Nitrogen (N) is one of the most important factors limiting plant productivity, and N fixation by legume species is an important source of N input into ecosystems. Meanwhile, N resorption from senescent plant tissues conserves nutrients taken up in the current season, which may alleviate ecosystem N limitation. N fixation was assessed by the ^15^N dilution technique in four types of alpine grasslands along the precipitation and soil nutrient gradients. The N resorption efficiency (NRE) was also measured in these alpine grasslands. The aboveground biomass in the alpine meadow was 4–6 times higher than in the alpine meadow steppe, alpine steppe, and alpine desert steppe. However, the proportion of legume species to community biomass in the alpine steppe and the alpine desert steppe was significantly higher than the proportion in the alpine meadow. N fixation by the legume plants in the alpine meadow was 0.236 g N/m^2^, which was significantly higher than N fixation in other alpine grasslands (0.041 to 0.089 g N/m^2^). The NRE in the alpine meadows was lower than in the other three alpine grasslands. Both the aboveground biomass and N fixation of the legume plants showed decreasing trends with the decline of precipitation and soil N gradients from east to west, while the NRE of alpine plants showed increasing trends along the gradients, which indicates that alpine plants enhance the NRE to adapt to the increasing droughts and nutrient‐poor environments. The opposite trends of N fixation and NRE along the precipitation and soil nutrient gradients indicate that alpine plants adapt to precipitation and soil nutrient limitation by promoting NRE (conservative nutrient use by alpine plants) rather than biological N fixation (open sources by legume plants) on the north Tibetan Plateau.

## Graphical abstract

The opposite trends of N fixation and N resorption efficiency along the precipitation and soil nutrient gradients indicate that alpine plants adapt to precipitation and soil nutrient limitation by promoting NRE (conservative nutrient use by nonlegume plants) rather than biological N fixation (open sources by legume plants) on the north Tibetan Plateau.

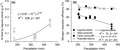

## 1 INTRODUCTION

Nitrogen (N) is one of the limiting nutrient elements to plant growth and productivity in many terrestrial ecosystems (Vitousek et al., 1997; Wedin & Tilman, 1996). It is also closely related to the composition, function, and stability of ecosystems (LeBauer & Treseder, 2008). As an important source of external N input into ecosystems, biological N fixation by symbiotic legume species is the process in which airborne N molecules in the atmosphere are reconstituted into a mixture of ammonia molecules, which play an important role in the terrestrial N cycle (Cusack, Silver, & McDowell, 2009; Vitousek et al., 2002). Biological N fixed rate by legume plants varies greatly among different ecosystems. Jacot, Lüscher, Nösberger, and Hartwig (2000) found that biological N fixation rate ranged from less than 1 g N m^−2^ year^−1^ in tundra ecosystems to 30 g N m^−2^ year^−1^ in legume agricultural ecosystems. Solheim, Zielke, Bjerke, and Rozema (2006) reported that annual N fixation in arctic tundra ecosystems ranged from 0.019 to 0.255 g N m^−2^ year^−1^, and averaged 0.127 g N m^−2^ year^−1^. The biological N fixation rates in a temperate grassland and an alpine meadow were 1.15 and 1.00 g N m^−2^ year^−1^, respectively (Yang, Qiao, Xu, & Ouyang, 2011). Biological N fixation is generally controlled by various environmental factors. Soil moisture is an important factor influencing N fixation due to its effect on plant and rhizobia growth. Generally, moderate soil moisture is beneficial for plant nutrient absorption and metabolic rates (Paul et al., 2003). However, drought soil conditions can lead to a decrease in biological N fixation by limiting plant root hair growth, rhizobium infections, and rhizobia proliferation (Yuan & Yang, 2003). Soil nutrient availability is another important factor influencing biological N fixation. A high N level may inhibit biological N fixation, and a moderate N addition level can promote plant growth and stimulate the formation of rhizobia nodules. In arid or semiarid ecosystems, rare rainfall is usually associated with low soil nutrient availability, which may affect plant nutrient use strategies (Cunningham, Summerhayes, & Westoby, 1999; Drury, Zhang, & Kay, 2003). Although the study of N fixation has been conducted in various ecosystems, the factors controlling N fixation need to be determined in different types of alpine grasslands.

Nutrient resorption is an important nutrient use strategy. Plants generally recover some mineral elements from their senescent leaves, save them in storage organs, and reuse them the following growing season. In this way, plants can alleviate the dependence on nutrient uptake from nutrient‐poor soil in the current growing season (Jiang et al., 2012; Lü et al., 2013). Nutrient resorption can also influence litter quality and its subsequent decomposition and nutrient cycling (Jiang et al., 2012; Lü et al., 2013). Environmental factors, especially those that have direct impacts on soil nutrient availability (such as soil water content), could affect leaf nutrient resorption efficiency (van Heerwaarden, Toet, & Aerts, 2003; Lü & Han, 2010). Evidence from grasslands (Lü & Han, 2010), bogs (van Heerwaarden et al., 2003), and forests (Li, Zheng, Han, Zheng, & Li, 2010) indicates that an increase in N availability generally reduces plant N resorption. Plants in nutrient‐poor soil may tend to resorb more N from their senescent tissues and leave little N in the litter for subsequent decomposition. In addition, plants in different functional groups may possess different nutrient resorption abilities. For example, legume species, those fixing atmospheric N through biological symbiosis, usually have lower N resorption efficiency relative to their high N content (Zhao et al., 2017). However, little is known about how the nutrient resorption efficiency of legume species varies along the gradient of soil water and nutrients compared with nonlegume species.

On the north Tibetan Plateau, precipitation decreases gradually from east to west, ranging from approximately 450 mm in the east to 50–80 mm in the west. Similarly, soil organic matter content decreases from 4.0% to <1.0% along the precipitation gradient (Zhao et al., 2017). Four types of alpine grassland ecosystems, that is, alpine meadow, alpine meadow steppe, alpine steppe, and desert steppe, are distributed and develop from east to west. Plant nutrient use strategies, such as nutrient acquisition and recycling, are important aspects of the N economics spectrum (Wright et al., 2004). In arid and semiarid alpine grasslands, soil water and soil nutrient availability are important factors influencing plant nutrient reuse and recycling (Lü & Han, 2010; Yuan et al., 2006). Plants that grow in nutrient‐poor environments often exhibit low productivity, small individuals, and slow growth rate (Grime, 1977). Therefore, the widely distributed legume species that can fix N from the atmosphere are important sources for natural N input to ecosystems. Little is known about the contribution of the N fixed by symbiotic legume plants in different types of alpine grasslands along the soil water and nutrient gradients. We aimed to answer the following questions: (a) How legume plants balance N fixation and N recovery through resorption, and (b) how symbiotic legume and nonlegume plants recover N from their senescent tissues to adapt to nutrient‐poor soil. We hypothesize that biological N fixation shows decreasing trends in the four alpine ecosystems from east to west along with the decline of precipitation. Meanwhile, nutrient resorption efficiency shows an increasing trend from east to west along with soil nutrient and precipitation decline on the north Tibetan Plateau. To test our hypotheses, we investigated biological N_2_ fixation by legume species using the ^15^N dilute technique in the four types of alpine ecosystems. In addition, we measured the N resorption of the abundant species in the four alpine ecosystems. Integrating the results, we reveal the N utilization strategies for legume and nonlegume species along the soil water and nutrient gradients on the north Tibetan Plateau.

## 2 MATERIALS AND METHODS

### 2.1 Experimental sites

The north Tibetan Plateau, also called the Changtang Plateau locally, lies in the northwest of the Tibet Autonomous Region, which is located between the Nyainqentanglha Mountains in the south and the Kunlun Mountains in the north as well as between 91°E of the Qinghai–Tibet highway in the east and the national boundary line in the west, covering approximately 6.0 × 10^5^ km^2^ (Wu, Shen, Zhang, & Shi, 2013; Zhao et al., 2017). The altitude ranges from 4,600 m to 5,100 m above sea level. As the harshest region in China, the Changtang Plateau is characterized as an arid climate zone with high evaporation. The annual potential evapotranspiration is more than 1,800 mm, and the arid index (defined as the annual potential evapotranspiration divided by annual precipitation) ranges from 1.6 to 20 (Mao, Lu, Zheng, & Zhang, 2006). The annual mean temperature is below 0°C, with the low value of –10°C in January and the high value of 14°C in July (Yang, Zhang, Miao, & Wei, 2003). In this region, there is an obvious precipitation gradient from east to west, with mean annual precipitation ranging from more than 450 mm in Nagqu to less than 100 mm in Ngari, of which 65%–85% falls from June to August (Wu et al., 2013; Zhao et al., 2017). Four types of alpine ecosystems are distributed and develop from east to west along the precipitation gradient, that is, alpine meadow (AM), alpine meadow steppe (AMS), alpine steppe (AS), and alpine desert steppe (ADS). The dominant plant species in AM is *Kobresia pygmaea*, with total community coverage more than 90% (Table 1)*.* Both AMS and AS are dominated by *Stipa purpurea*, while some sedge plants, such as also *Carex capillifolia*, occurred in AMS. The total community coverage in AMS and AS is 30%–40%. *S. purpurea* and *Oxytropis microphylla* are dominant plant species in ADS, with total community coverage approximately 20%–30% (Zong, Zhao, & Shi, 2019). Soils are characterized as alpine frost calcic soil and desert soil (Zhao et al., 2017).

**Table 1:** Basic information in the study sites

	Study site			
	Alpine meadow in Nagqu	Alpine meadow steppe in Bangoin	Alpine steppe in Nyima	Alpine desert steppe in Gerze
Location	31°34′N 92°34′E	31°23′N 90°14′E	31°47′N 87°14′E	32°22′N 82°16′E
Altitude (m)	4,570	4,590	4,580	4,520
MAP (mm)	444.9	335.4	327.4	175.2
MAT (°C)	−0.9	−1.0	−1.4	−1.4
SWC (%)	8.40a	1.45c	4.43b	2.15c
STN (%)	0.364a	0.184b	0.151b	0.149b
STP (%)	0.0225b	0.0230b	0.0256b	0.0352a
Grassland types	Alpine meadow	Alpine meadow steppe	Alpine steppe	Alpine desert steppe
Dominated species	*Kobresia pygmaea*	*Stipa purpurea*	*S. purpurea*	*S. purpurea*, *Oxytropis microphylla*
Legume plants	*Stracheya tibetica* Benth.	*Oxytropis stracheyana* Benth. ex Baker, *Astragalus confertus* Benth. ex Bunge	*A. confertus* Benth. ex Bunge	*O. microphylla*, *A. confertus* Benth. ex Bunge

Note

MAP, MAT, SWC, STN, and STP represent precipitation, mean annual temperature, soil water content, and soil total N content and P content, respectively. Different lowercase letters following SWC, STN, and STP represent significant differences among the four study sites.

### 2.2 Experimental design and treatment

A biological N fixation experiment was conducted in early August 2015. Along the precipitation gradient from east to west, four alpine grasslands, that is, alpine meadow, alpine meadow steppe, alpine steppe, and alpine desert steppe located in Nagqu (NQ), Bangor (BG), Nyima (NM), and Gerze (GZ), respectively, were selected to for the labeling experiment (Table 1). Ten 1 × 1 m^2^ plots were randomly set up in each of the four alpine grasslands that were excluded from livestock grazing. (^15^NH_4_)_2_SO_4_ (98.4% ^15^N enrichment) was dissolved in pure water and sprayed in five plots with an N addition rate 0.03 g N/m^2^ in early August 2015, and the remaining five plots were sprayed pure water as the control. This N addition rate is far less than the level of soil available N in soil and cannot significantly affect soil nutrient content or plant nutrient uptake (Yang et al., 2011). The total amount of water used in the labeling equal to 2.0 mm of precipitation.

### 2.3 Biological N fixation

We used the ^15^N dilution technique to quantify N fixation by legume plants. An important assumption is that the reference plants accumulate N with the same amount of ^15^N enrichment from soil as legume plants (Lonati, Probo, Gorlier, & Lombardi, 2015; Yang et al., 2011). In this study, we selected a graminoid *S. purpurea* as the reference plant. Reference plants often affect the estimation of N fixation when the ^15^N dilution technique is used (Lonati et al., 2015; Yang et al., 2011). The reason is because both legume species and graminoid *S. purpurea* are perennial. Meanwhile, only 4–5 species occur in alpine ecosystems, and even fewer species occur in the alpine desert steppe. *S. purpurea* is the only species simultaneously occurring in the four study sites. We used the same species as a reference plant in the four ecosystems which make the results comparable among ecosystems. In addition, a previous study showed that reference species from different functional groups, such as graminoids or nongraminoids, had little effect on the results (1.00 vs. 0.91 g N m^−2^ year^−1^ in an alpine grassland and 1.15 vs. 1.16 g N m^−2^ year^−1^ in a temperate grassland; Yang et al., 2011). Thus, the estimation method of biological N fixation is reasonable and comparable in different alpine grasslands on the Changtang Plateau.

One month after labeling, all the aboveground plant materials in the labeled and unlabeled plots were collected in the 1.0 × 1.0 m^2^ plots in early September 2015. The plant samples collected for ^15^N analysis were mainly in the center of each 1 × 1 m^2^ plot to avoid the edge effect (at least 15 cm from the edge of the plot). Each species was separately clipped at the ground level, then oven‐dried at 65°C for over 48 hr in laboratory, and weighed for aboveground biomass. Five soil cores were randomly sampled in each plot using an auger (3.8 cm in diameter and 20 cm in depth) after plant material collection. The five cores from each quadrat were mixed together as one composite sample. Soil samples were passed through a 2‐mm diameter soil sieve, and visible living plant roots were discarded. The sieved soil was used for further laboratory measurement. The soil total N content was determined by the Kjeldahl method.

The collected plant materials were put into a 0.5 mol/L CaCl_2_ solution for 30 min to remove the N residue on the plant leaves and oven‐dried at 65°C for 48 hr in the laboratory. All the plant materials were ground to a fine powder using a ball mill (MM200, Fa. Retsch). N content and the ^15^N/^14^N ratio were measured with a MAT 253 stable isotope ratio mass spectrometer system (MAT 253, Finnigan MAT).

The stable isotope abundances were calculated as follows:

$$\delta^{15}N=\left(\frac{R_{\text{sample}}}{R_{\text{standard}}}-1\right)\times 1000$$

where *R* is the ratio of ^15^N/^14^N either in the sample or in the standard sample.

Atom% excess ^15^N (APE) was calculated as the atom %^15^N difference between the same plant species from the ^15^N‐treated (APE_Labeled_) and the control plots (APE_Nonlabeled_).

$$\text{APE}={\text{APE}}_{\text{Labeled}}-{\text{APE}}_{\text{Nonlabeled}}$$

We used the following equation to calculate N derived from the atmosphere (%Ndfa) for the legume species (Lonati et al., 2015; Yang et al., 2011). The calculations used the APE of the legume species and the reference plants in the same site.

$$\%\text{Ndfa}=\left(1-\frac{{\text{APE}}_{\text{Legume}}}{{\text{APE}}_{\text{Reference}}}\right)\times 100$$

Legume plant biomass was used to estimate the amount of N fixed by the legume species of per unit area. The quantity of N fixed by the legume species (N fixed, g N/m^2^) was calculated using the following equation (Busse, 2000):

$${\text{N fixed}}_{\text{area}}=\left(\%\text{Ndfa}\right)/100\times \text{TN}/100\times \text{biomass}$$

where TN is the N content in the plant materials.

### 2.4 Nitrogen resorption efficiency

Most alpine plants in this region start to grow in May and senesce in late September with a peak growing season from late July to early August (Wu et al., 2014). Mature and senescent plant leaves were collected from alpine deserts, steppes, steppe meadows, and meadows across the Changtang Plateau in late July and early October 2014, respectively. We chose four plant species that are abundant in each functional group, that is, *Oxytropis* sp. (legume species), *S. purpurea* (grasses), *C. moorcroftii* (sedges), and *Potentilla bifurca* L. (forbs), to represent the change in the plant communities (Zhao et al., 2017). At each site, at least 20 plant individuals with mature and fully extended leaves (Jiang et al., 2012; Zhao et al., 2017) were randomly selected with five replicate sites at 500‐m intervals in late July and early October, respectively. The criteria used for the determination of the senescent leaves was followed in the reference Jiang et al. (2012), which was that recently senesced, brown, but still attached leaves were collected in each plot. Soil samples at a depth of 0–20 cm were collected from each study site in late July.

Plant samples were oven‐dried at 65°C for 48 hr to a constant weight and ground using a mill (MM200, Fa. Retsch). Air‐dried soil samples were also ground. The leaf N and soil total N content were measured using a C/N analyzer (Elementar vario MAX).

Nitrogen resorption efficiency (NRE) was calculated as follows:

$$\text{NRE}=\frac{N_{\text{mature}}-N_{\text{senesced}}\times \text{MLCF}}{N_{\text{mature}}}\times 100\%$$

in which *N*_mature_ and *N*_senesced_ are the N content in the mature and the senescent leaves, respectively. MLCF represents the mass loss correction factor, which was used to compensate leaf mass loss during the senescent process (Van Heerwaarden et al., 2003). The values of MLCF were 0.713 for herb plant species (Vergutz, Manzoni, Porporato, Novais, & Jackson, 2012). The NRE of each plant species in the same grassland type was averaged as the NRE for each grassland type.

### 2.5 Statistical analysis

One‐way ANOVA followed by Duncan's multiple comparison was used to detect the difference in the aboveground biomass of the community, the legume and nonlegume plants, and the proportion of legume plants to the community biomass in the four types of alpine ecosystems. One‐way ANOVA was also used to analyze the differences in N content of the legume and nonlegume plant leaves, the soil total N content, the %Ndfa values, and N fixation in the four types of alpine ecosystems. Regression analysis was used to analyze the correlations of precipitation with N fixation and NRE. A general linear model was used to test the significance of the correlation slope between the legume and nonlegume plants. Linear regression was used to analyze the relationships of N fixation and biomass of the legume plants with the soil N content as well as the relationship of NRE with N content in green leaves and in soil. Statistical significance was *p* < .05. All statistical analyses were performed using the SPSS 16.0 software package (SPSS). All figures were produced using Origin Pro 8.0 (OriginLab Corporation).

## 3 RESULTS

### 3.1 Aboveground biomass and biological N fixation in different alpine grasslands

**Figure 1:**
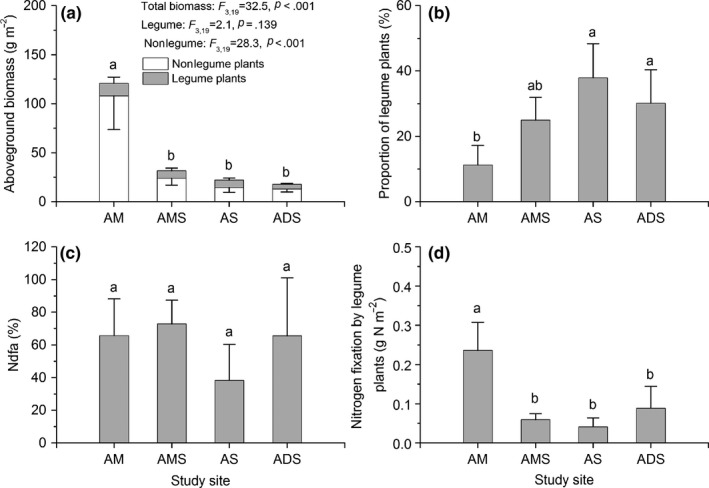
Aboveground biomass of legume and nonlegume plants (a), and the proportion of legume biomass to total community (b) in different types of alpine grassland. %Ndfa and the N fixed from the atmosphere by legume plants and in different alpine grasslands are shown in c and d, respectively. Values are mean ± *SE* (*n* = 5). Four alpine grasslands, that is, alpine meadow (AM), alpine meadow steppe (AMS), alpine steppe (AS), and desert steppe (ADS), were represented by the location in Nagqu (NQ), Bangor (BG), Nyima (NM), and Gerze (GZ), respectively. Different letters mean significant differences among different alpine grasslands at *p* < .05 (Duncan's test). The same is below

The aboveground biomass was significantly different among the four types of alpine ecosystems (Figure 1). The biomass in the alpine meadow was four to six times higher than in other three types of ecosystems (Figure 1a, *p* < .001). The biomass did not differ among the other three ecosystems. Moreover, the aboveground biomass of the legume plants was not significantly different among the four alpine ecosystems (Figure 1a, *p* = .139). The ratio of legume biomass to total community biomass ranged from 11% to 37%, and this ratio was significantly higher in the alpine steppe and the alpine desert steppe than in the alpine meadow (Figure 1b, *p* = .029).

The atom% excess ^15^N in nonlabeled legumes ranged from 1.11‰ to 2.53‰, while this value in nonlabeled reference plants was from 1.39‰ to 2.06‰ (Table 2). Atom% excess ^15^N in labeled plants was higher than those in nonlabeled plants. The atom% excess ^15^N in labeled legumes ranged from 1.21‰ to 2.74‰, while this value in labeled reference plants was from 1.67‰ to 2.42‰ (Table 2). The %Ndfa values were not significantly different among the alpine grasslands, ranging from 38% to 72% (Figure 1c), indicating that the biological N fixation ability did not differ in different grasslands along the precipitation and soil nutrient gradients on the Changtang Plateau. N fixation by the legume plants was higher in the alpine meadow (0.236 g N/m^2^) than in the other three types of alpine ecosystems (Figure 1d). N fixation was not significantly different among the other three types of alpine ecosystems, ranging from 0.064 to 0.095 g N/m^2^ (Figure 1d).

**Table 2:** Atom% excess ^15^N (APE) in labeled and nonlabeled legume and nonlegume plants (‰), and the subtraction of labeled to nonlabeled APE in different plant species

Study sites	Legume species		Reference plants		Subtraction of labeled to nonlabeled APE	
	Labeled	Nonlabeled	Labeled	Nonlabeled	APE_Legume_	APE_Reference_
Alpine meadow in Nagqu	2.66 (0.02)	2.44 (0.11)	1.99 (0.12)	1.47 (0.12)	0.21(0.18)	0.52 (0.20)
Alpine meadow steppe in Bangoin	1.21 (0.12)	1.11 (0.17)	1.67 (0.15)	1.39 (0.14)	0.09 (0.07)	0.27 (0.11)
Alpine steppe in Nyima	2.74 (0.09)	2.53 (0.19)	2.42 (0.16)	2.06 (0.13)	0.21 (0.09)	0.36 (0.21)
Alpine desert steppe in Gerze	2.61 (0.14)	2.45 (0.08)	2.37 (0.05)	2.00 (0.05)	0.16 (0.17)	0.37 (0.09)

Note

The data in the brackets represent the standard error. The subtraction of labeled to nonlabeled APE (APE_Legume_ and APE_Reference_) represents the differences in atom% excess ^15^N in the labeled plant species subtracts the nonlabeled in legume species and reference plants, respectively.

### 3.2 Nitrogen resorption in different alpine grasslands

**Figure 2:**
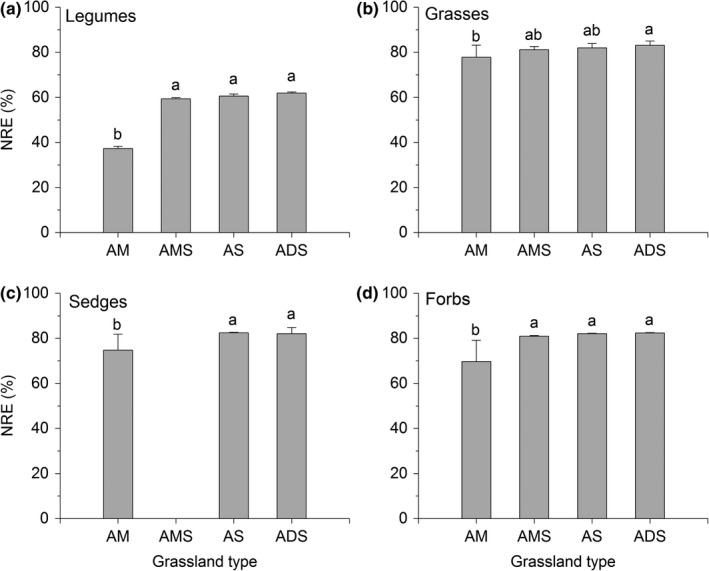
Nitrogen resorption efficiencies of the legume species (a) and the dominant plant species (b–d) in the alpine grasslands

Nitrogen content in plant mature leaves ranged from 2.022% to 3.926%, and these values in legume plants were generally higher than those in other plant functional groups (Table 3). N content in plant senescent leaves ranged from 0.603% to 3.267%, and the corrected N content in plant senescent leaves was ranging from 0.430% to 2.329%. The N resorption efficiency showed similar trends among the four functional groups (Figure 2, Table 3). The NRE was lower in the alpine meadow than in the alpine meadow steppe, the alpine steppe, and the alpine desert steppe, with exception that the NRE of grasses was not significantly different in the alpine meadow as compared with the NREs of the alpine meadow steppe and the alpine steppe (Figure 2, *p* = .063).

**Table 3:** Nitrogen content in plant mature and senescent leaves as well as the nitrogen resorption efficiency (NRE) of different plant functional groups in different study sites

Study sites	Plant functional groups	*N*_mature_ (%)	*N*_senesced_ (%)	Corrected *N*_senesced_ (%)	NRE (%)
Alpine meadow in Nagqu	Legumes	3.713 (0.072)	3.267 (0.000)	2.329 (0.000)	37.26 (0.10)
	Grasses	2.022 (0.166)	0.641 (0.211)	0.457 (0.150)	77.80(5.40)
	Sedges	2.542 (0.432)	0.916 (0.341)	0.653 (0.243)	74.79 (7.08)
	Forbs	2.385 (0.167)	1.002 (0.282)	0.714 (0.201)	69.69 (9.48)
Alpine meadow steppe in Bangoin	Legumes	3.926 (0.163)	2.238 (0.060)	1.596 (0.043)	59.34 (0.49)
	Grasses	2.298 (0.309)	0.603 (0.055)	0.430 (0.039)	81.15 (1.46)
	Sedges	—	—	—	—
	Forbs	2.529 (0.033)	0.675 (0.006)	0.481 (0.004)	80.96 (0.34)
Alpine steppe in Nyima	Legumes	3.778 (0.311)	2.087 (0.109)	1.488 (0.078)	60.55 (0.97)
	Grasses	2.793 (0.373)	0.709 (0.140)	0.506 (0.099)	81.95 (1.93)
	Sedges	3.132 (0.123)	0.772 (0.018)	0.550 (0.013)	82.43 (0.24)
	Forbs	3.155 (0.45)	0.795 (0.000)	0.567 (0.000)	82.03 (0.36)
Alpine desert steppe in Gerze	Legumes	3.115 (0.082)	1.664 (0.014)	1.186 (0.009)	61.90 (0.55)
	Grasses	2.679 (0.149)	0.632 (0.046)	0.451 (0.033)	83.08 (1.90)
	Sedges	2.481 (0.109)	0.622 (0.090)	0.443 (0.064)	82.06 (2.75)
	Forbs	3.072 (0.276)	0.760 (0.057)	0.542 (0.041)	82.35 (0.23)

Note

“—” represents no data in this study site. The data in the brackets represent the standard error.

### 3.3 Correlations of precipitation and soil N content with N fixation and N resorption

**Figure 3:**
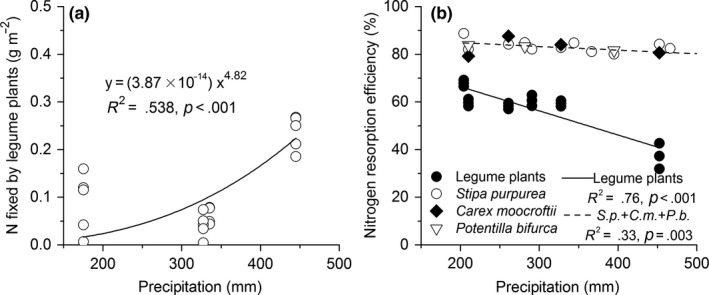
Relationships of N fixed by legume plants (a) and N resorption efficiency (b) with the precipitation along the Changtang grassland transect. All the plants were sorted into legume and nonlegume plants. The nonlegume species are *Stipa purpurea* (*S.p.*)*, Carex moocroftii* (*C.m.*), and *Potentilla bifurca* (*P.b.*)

N fixation by the legume plants showed a positive exponential trend with varying precipitation from east to west (Figure 3a, *p* < .001), indicating that precipitation on the Changtang Plateau is an important factor controlling biological N fixation in grasslands. The NRE of both the legume and nonlegume plants showed significantly negative correlations with precipitation, and the slope was significantly lower for the legume plants than that for the nonlegume plants (Figure 3b; *F* = 121.32, *p* < .001).

### 3.4 Relationships of N concentration with N fixation and N resorption

**Figure 4:**
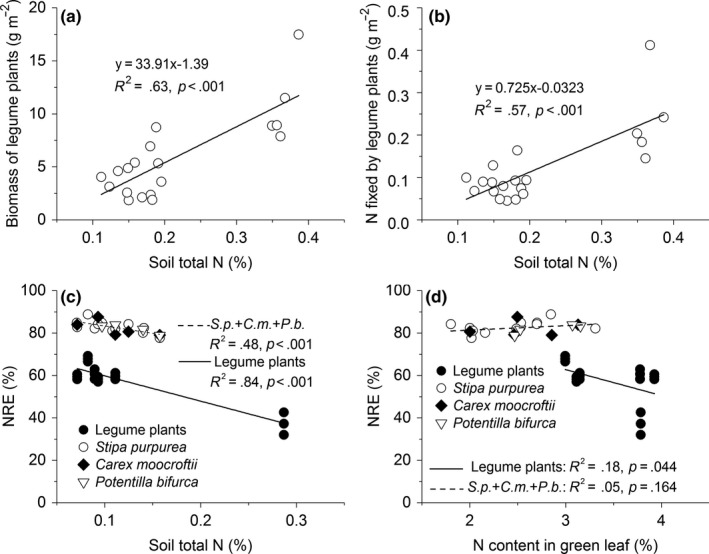
Relationships of legume plant biomass (a), N fixed by legume plants (b), and N resorption efficiency (c) with soil total N content, as well as relationship between N resorption efficiency (NRE) and green leaf N concentration (d). All the plants were sorted into legume and nonlegume plants. The nonlegume species are *Stipa purpurea* (*S.p.*)*, Carex moocroftii* (*C.m.*), and *Potentilla bifurca* (*P.b.*)

The soil N concentration showed a decreasing trend from east to west, and the N concentration was significantly higher in the alpine meadow than in the other three alpine ecosystems (Table 1, *p* < .05). No significant difference in N content was found between the alpine steppe and the desert steppe (Table 1, *p* > .05). The soil N content was positively correlated with the biomass of the legume plants and the biological N fixation (Figure 4a,b, *p* < .001). However, the NRE of nonlegume species was negatively correlated with soil N content, but was not correlated with the N content of the plant green leaves (Figure 4c,d). Both soil N and N content in the plant green leaves had significant correlations with the NRE of legume plants (Figure 4c,d).

## 4 DISCUSSION

In most natural ecosystems, N fixation by legume species is the most important source of N input from the atmosphere. Our results illustrate that N derived from the atmosphere (%Ndfa) did not differ in the four types of alpine ecosystems, while biological N fixation displayed a decreasing trend with precipitation decline, which is partly consistent with the first hypothesis. The high amount of N fixation in the alpine meadow was mainly attributed to high plant biomass. However, N resorption efficiency in the alpine species displayed an increasing trend along with a decline in soil nutrients and precipitation from east to west on the Changtang Plateau, which supports our second hypothesis. As previously mentioned, biological N fixation and NRE displayed opposite trends along the precipitation gradient on the Changtang Plateau, indicating that alpine plants show conservative nutrient use strategies from wet to arid environments. That is, they conserve N by increasing nutrient resorption rather than obtaining N from open sources through symbiotic fixation.

### 4.1 Biological N fixation and its driving factors in alpine grasslands

The %Ndfa value represents the proportion of the N derived from air to the total N content in legume plants. In this study, the %Ndfa values ranged from 38% to 72% in the alpine grasslands along the precipitation gradient. The previous studies showed %Ndfa values ranging from 59% to 90% in the Swiss Alps (Jacot et al., 2000) and from 50.4% to 89.7% in an alpine meadow on the Tibetan Plateau (Yang et al., 2011). The %Ndfa values in our study were within these ranges, indicating that N fixation by legume plants on the Changtang Plateau is similar to the abovementioned ecosystems.

In our study, the total aboveground N fixation by the legume species was 0.236 g N/m^2^ in the alpine meadow, which is lower than the 1.0–2.6 g N/m^2^ reported in Swiss Alps (Jacot et al., 2000) and the 1.00 g N/m^2^ and 1.15 g N/m^2^ reported in a wet alpine meadow and a temperate grassland, respectively (Yang et al., 2011). Moreover, the total aboveground N fixation was 0.041 g N/m^2^ and 0.089 g N/m^2^ in the alpine meadow steppe and the alpine steppe, respectively, which is also lower than the 0.25 g N/m^2^ reported in alpine steppe (Dai, 2015) and 0.49 g N/m^2^ reported in a wet alpine meadow (Bowman, Schardt, & Schmidt, 1996). The reason may mainly be ascribed to the higher community biomass in the other ecosystems in the previous studies. In our study, the aboveground biomass was 107 g/m^2^ in the alpine meadow, 23 g/m^2^ in the alpine steppe, and 14 g/m^2^ in the alpine desert community. The biomass of the legume plants was only 4.9–12.9 g/m^2^ from the alpine meadow to the alpine desert steppe. The low biomass is mainly due to the low temperatures and the poor soil nutrients in the high altitude plateau. However, the biomass of the legume plants was 48.4 g/m^2^ and 45.9 g/m^2^ in a wet alpine meadow and a temperate grassland, respectively (Yang et al., 2011), nearly ten times higher than the biomass in our study. In addition, the biomass amounted to 2.6 g N/m^2^ in the Swiss Alps (Jacot et al., 2000), which is higher than this estimation. The lower altitude and higher temperature could be the explanation for the almost ten times higher N fixation reported in the Swiss Alps. Although Dai (2015) conducted a similar study in an alpine steppe, the precipitation (335 mm) and plant aboveground biomass (144.5 g/m^2^) were comparable with the alpine meadow in our study. Moreover, aboveground N fixation was also similar to the value in the alpine meadow in our study. Furthermore, N fixation in our study (0.041–0.089 g N m^−2^ year^−1^) was comparable to the N input by precipitation in remote sites (0.044 g m^−2^ year^−1^ in Ngari and 0.092 g m^−2^ year^−1^ in Nam Co; Liu, Xu, Wang, Pan, & Piao, 2015), so the contribution of biological N to overall ecosystems should not be ignored.

The higher N fixation in the alpine meadow than in the other three alpine ecosystems supports our hypothesis. Along the transect, soil N content was positively exponentially correlated with precipitation (Zhao et al., 2017), indicating that soil N availability was improved with increasing precipitation. We also found that N fixation in alpine grasslands was exponentially correlated with precipitation along the Changtang transect. Soil moisture is a key factor regulating plant growth and metabolic activity in arid and semiarid regions (Niu et al., 2008), and moderate soil moisture is generally beneficial for soil nutrient absorption and microbial activity. The number of rhizobia in appropriate soil moisture conditions was significantly higher than that in soil conditions that were too wet or too dry (Chalk & Smith, 1997). Thus, the decreasing legume biomass may contribute to the decreased N fixation along the soil water content gradient from east to west. The increase in soil N content was ascribed to the enhancement of plant biomass from west to east. We concluded that precipitation can directly affect the N fixation of legume plants by influencing their growth, and it can also indirectly affect N fixation by controlling soil N availability.

More than 95% of the total N in alpine meadow soils is organic N, while only approximately 1% of N is inorganic N (Zhou, 2001). A previous study reported that alpine plants can directly absorb soil organic nitrogen (mainly in free amino acids) in arctic tundra and alpine meadow ecosystems (Kielland, 1994; Wang et al., 2012). In this study, we only used (^15^NH_4_)_2_SO_4_ (98.4% ^15^N enrichment) to estimate the amount of N fixation by legume plants in alpine grasslands. If legume plants in alpine meadow absorb nitrate or DON (not labeled by ^15^N) in soil, it might cause similar isotopic changes even without increasing N fixation. A study indicated that the amount of N fixation by legume plants had some differences when different forms of N (K^15^NO_3_ and ^15^NH_4_Cl) were used in the labeling experiment due to the absorptive preference of different forms of N by different reference plants (Wang et al., 2012). Thus, special attention should be paid when different plants are used to estimate the amount of N fixation in further studies.

### 4.2 Nitrogen resorption and use strategies of legume and nonlegume species

As a nutrient conservation strategy, nutrient resorption from senescent leaves is an important internal nutrient recycling process and a conservative mechanism for plants to adapt to nutrient deficiency and increase their fitness, particularly in nutrient‐poor environments, such as alpine and arid ecosystems (Aerts, 1996). In our study, the corrected N resorption efficiencies of the legume and nonlegume plants were 37.3%–61.9% and 70.0%–83.1%, respectively. The leaf N resorption efficiency in the legume species was significantly lower than that of the nonlegume species. The low N resorption efficiency of legume species may be attributed to their lower dependence on soil available N, which is compensated by their N fixation ability from the atmosphere. We also found that the N resorption efficiency in the nonlegume species was negatively correlated with soil N content along the precipitation gradient, consistent with the results from other manipulative fertilization experiments (Huang et al., 2008; Lü et al., 2013). In addition, we found that leaf N resorption efficiency displayed a descending trend with the increase in precipitation from west to east, which is consistent with other studies (Meier & Leuschner, 2014; Reed, Townsend, Davidson, & Cleveland, 2012). At regional and local scales, precipitation is a key factor affecting soil moisture and fertility, regulating plant nutrient conditions and resorption (Brant & Chen, 2015), and it plays an important role in driving biogeochemical cycles in arid or semiarid ecosystems (Austin et al., 2004; Schwinning & Sala, 2004). Therefore, in a nutrient‐limited environment, alpine plants are likely to evolve specific conservative adaptation strategies to low nutrient stress.

Plant species usually take use strategies to alleviate nutrient limitation either by promoting biological N fixation (an opening nutrient source) or by increasing their nutrient resorption efficiency (reducing nutrient loss) in soils with poor nutrients (Jaeger & Monson, 1992). The differences in N fixation were mainly attributed to plant production and leaf N content. In addition, although legume species can directly fix N from the atmosphere, they still partly resorb N from senescent leaves, especially in the alpine desert steppe in arid areas (Figure 3). This indicates that legume plant species still show a conservative nutrient use strategy, although the NRE in the legume species was lower than in nonlegume species. The nonlegume plant species have an even higher NRE than the legume species, indicating that they are more dependent on nutrient resorption because they cannot fix N directly. The NRE in both the legume and the nonlegume species was negatively correlated with precipitation, while the biological N fixation of the legume species displayed a positive trend along the precipitation gradient on the Changtang Plateau. It seems that there exists a trade‐off between resource acquisition and nutrient retention (Wright et al., 2004). In a nutrient‐limited environment, plants generally show a slow growth rate and conservative nutrient use, which is characterized by a high C/N ratio, a high leaf nutrient resorption efficiency, and a low litter decomposition rate (Aerts & Chapin, 2000; De Deyn, Cornelissen, & Bardgett, 2008; Orwin et al., 2010). Our results suggest that alpine plants adopt conservative nutrient use strategies from wet to arid environments, that is, they conserve N by increasing leaf nutrient resorption rather than obtaining N from open sources through symbiotic N fixation.

It is reported that leaf size change and mass loss may lead to considerable underestimation of resorption. Leaf shrinkage could result in an average underestimation of 6% when using area‐based concentrations (Van Heerwaarden et al., 2003). Previous study along this transect reported that the size of the leaves during abscission may not change much in the arid and semiarid climate and the amount of carbon loss in senesced leaves was small with an average less than 0.2% (Zhao et al., 2017). We also used the corrected method to recalculate the N content in senesced leaves according to the mass loss correction factor, which was used to compensate leaf mass loss during the senescence period (Van Heerwaarden et al., 2003; Vergutz et al., 2012). Based on the recorrection, the resorption efficiencies for N and P (except legume plants) were higher than the corrected world means of N (62.1%) and P (64.9%), respectively (Vergutz et al., 2012). Therefore, leaf shrinkage and mass loss deserve to be considered in order to avoid real estimation of resorption efficiency in the future study. This also suggests that the alpine plants in this study could have even higher resorption efficiencies than other regions, and unravels the fact of nutrient resorption higher on the Plateau than the world average and further demonstrates the growth of alpine plants is highly limited by soil nutrient availability.

### 4.3 Importance of legumes in alpine grasslands

In our study, N fixation in the alpine meadow (0.236 g N/m^2^) was 4.7 times higher than N fixation in the alpine steppe (0.050 g N/m^2^, including the alpine meadow steppe and the steppe), and 2.7 times higher than N fixation in the alpine desert (0.089 g N/m^2^). Legume plants are widely distributed across the grassland ecosystems on the Tibetan Plateau, and they are generally considered as poisonous weeds due to their toxicity and inedibility for livestock (Wu, Yang, Zhang, Shen, & Yu, 2015). However, N fixed by the legume plants is a key N input directly from the atmosphere in natural ecosystems, and their contribution to soil N should not be ignored.

Alpine meadow, alpine steppe, and alpine desert steppe are the three main grasslands on the Tibetan Plateau, with areas of 1.873 × 10^7^, 4.958 × 10^7^, and 0.025 × 10^7^ ha, respectively (Wang, Qian, Cheng, & Lai, 2002). Based on the amount of N fixed by the legume plants in our study, the total aboveground N fixation was approximately 4.4 × 10^–2^, 2.5 × 10^–2^, and 2.23 × 10^–4^ Tg N/year in the alpine meadow, the alpine steppe, and the alpine desert steppe, respectively, on the Tibetan Plateau. The belowground biomass is much higher than the aboveground biomass in alpine grasslands on the Tibetan Plateau, and belowground‐to‐aboveground biomass ratios were 9.0, 3.8, and 6.1 in the alpine meadow, the alpine steppe, and the alpine desert steppe, respectively (Zeng, Wu, & Zhang, 2015). N fixation in belowground tissue should be assessed in further studies. Given that legume plants are widely distributed in different alpine grasslands on the Tibetan Plateau, the amount of N fixation could be of great importance in the ecosystem N cycle and for plant production maintenance.

## CONFLICT OF INTEREST

The authors declare that they have no conflict of interest.

## AUTHOR CONTRIBUTIONS

NZ and PLS designed the experiments. NZ and GSZ performed the experiments. NZ, GSZ, and MHS analyzed the data. NZ wrote the manuscript. NZ, MHS, and PLS revised the manuscript. All authors read and approved the final manuscript.

## Data Availability

Data are available from the Dryad Digital Repository: https://doi.org/10.5061/dryad.x3ffbg7f8.
